# Oculomotor deficits in attention deficit hyperactivity disorder: a systematic review and meta-analysis

**DOI:** 10.1038/s41433-022-02284-z

**Published:** 2022-10-24

**Authors:** Sharath S. Sherigar, Ashwitha H. Gamsa, Krithica Srinivasan

**Affiliations:** Department of Optometry, Manipal College of Health Professions (MCHP), Manipal Academy of Higher Education (MAHE), Manipal, Karnataka India

**Keywords:** Visual system, Ocular motility disorders

## Abstract

There is equivocal evidence on the presence of oculomotor deficits among children with attention deficit hyperactivity disorder (ADHD), which can be an additional challenge in this population, especially with reading-related tasks. This study aimed to review the deficits in the oculomotor parameters among children with ADHD compared with age-matched controls. The review was conducted according to the Preferred Reporting Items for Systematic Reviews and Meta-analyses (PRISMA) guidelines. A search of original research articles on various databases was done using key terms, such as “oculomotor deficit,” “attention deficit hyperactivity disorder,” or related terms. We included case-control studies and excluded studies in which children received medications during the test. Twelve original research studies were considered for this review. Ten studies reported data on various types of saccades, two studies reported data on fixation, and one study reported data on pursuit. Among various oculomotor deficits, the forest-plot analysis of an antisaccade task showed that children with ADHD made more direction errors compared to controls. Although independent studies report that ADHD children have poorer performance compared to control populations during oculomotor tasks, there is a lack of evidence to draw a strong conclusion. Children with ADHD are less precise in performing eye movements and need more time to complete the oculomotor tasks than those without ADHD. The overall results provide minimal evidence regarding the presence of various oculomotor deficits in ADHD.

## Abstract (zh)

多动障碍缺陷 (ADHD) 儿童存在动眼神经缺陷的证据尚不明确, 尤其是阅读相关的活动, 对他们来说可能是额外的挑战。本研究旨在回顾与年龄匹配的对照组相比, ADHD儿童动眼神经参数方面的缺陷。该综述是根据系统综述和荟萃分析 (PRISMA) 的指南进行的。我们使用关键词如“动眼神经缺陷”、“注意力多动障碍缺陷”或相关术语对各种数据库中的研究文献进行了搜索 。纳入了病例对照研究, 排除了在试验间接受药物治疗的儿童研究。

本综述纳入了12项原始研究。十项研究报告了各种类型的扫视数据, 两项研究报告的是注视数据, 一项研究报告是追视数据。在各种动眼神经缺陷中, 相反扫视任务的森林图分析表明, 与对照组相比, ADHD儿童出现更多的方向错误。尽管独立研究报告表明ADHD儿童在动眼神经任务中的表现比对照组差, 但缺乏证据得出强有力的结论即ADHD儿童在进行眼球运动时不太精准, 需要比正常儿童花费更多的时间完成动眼神经任务。本研究的结果提供了关于ADHD中存在各种动眼神经缺陷的微弱证据。

## Introduction

Neurobehavioral disorders characterised by the lack of attention, impulsivity, and extremely active and disruptive behaviour in childhood or adulthood are referred to as attention deficit hyperactivity disorder (ADHD) [1–3]. ADHD is also termed hyperkinetic disorder according to the World Health Organisation classification system [4]. The prevalence of ADHD globally is around 2.2% among children and adolescents aged <18 years [5].

Oculomotor behaviour involves saccades, smooth pursuit, and fixations [6]. In the basal ganglia, superior colliculus, cerebral cortex, cerebellum and thalamus, the oculomotor systems have a widely distributed network [7]. Neuroimaging studies in ADHD have shown a reduction in the volume of frontal regions, cerebellum and sensorimotor brain regions [8] and reduced activation in the basal ganglia and anterior frontal cortex [9]. These findings support the aetiology of oculomotor deficits reported in ADHD [10]. Oculomotor skills can be assessed based on fixation and eye movements pattern [11]. Motor skill deficiency is widespread among ADHD patients due to abnormal development of cerebellar-frontostriatal circuits related to this disorder [10]. Children with ADHD experience difficulties in tasks requiring complex abilities, known as executive function [12]. Apart from oculomotor deficits, such as poor motor skills, effortful processing in the routine tasks, slower processing speed, and lack of attention have also been reported in ADHD [10]. The presence of all these deficits correlates with the motor and executive malfunction reported in ADHD [10].

Various eye-tracking studies have shown abnormal eye movements in ADHD [13, 14]. The variables obtained from the oculomotor paradigms capture three critical factors of executive control, including response inhibition (antisaccade and anticipatory errors), working memory (memory-guided saccade (MGS)) and response preparation (saccade latency and variability) [10]. These eye-tracking/movement tasks are painless, objective, and non-invasive methods, providing insight into the underlying neurophysiology of disease [15]. The evaluation of these eye movement tasks is important in assessing the integrity of pathways of the brain in healthy and diseased conditions [16]. In case of abnormal eye movements, the eye-tracking task helps explore the specific aspects of the oculomotor control on various behavioural tasks among ADHD patients [13, 14]. There are maturational abnormalities in brain systems seen in neurodevelopmental disorders, and the oculomotor model is a promising method for its characterisation [17]. The presence of oculomotor deficits leads to significant difficulties among younger children and necessitates minor adaptation in school [18]. In this systematic review, we aimed to summarise the deficits in oculomotor functions, such as saccade, smooth pursuit and fixation among children with ADHD.

## Materials/subjects and methods

The review was conducted according to the Preferred Reporting Items for Systematic Reviews and Meta-analyses (PRISMA) guidelines [19]. The review was registered in PROSPERO, with the registration number CRD42020209319.

### Review question

The aim of this review was to compare the oculomotor parameters in children with and without ADHD. Outcome measures of this review were oculomotor parameters, such as saccades, pursuits and fixation deficits.

### Search strategy

A literature search to identify the publications relevant to the review was done using various electronic databases, such as PubMed, PMC (MEDLINE), Web of Science, Scopus, CINHAL complete, and BMJ. The search terms included “saccade,” “smooth pursuit,” “oculomotor deficit,” “oculomotor problem,” “oculomotor deficit,” “fixation” and “attention hyperactivity disorder.” During the search, appropriate Boolean operators, such as “OR,” “AND” and “NOT,” were used, including Medical Subject Heading (MeSH) terms, to identify the relevant literature. The list of the search strategy used is provided in the Supplemental Material. All references were imported and managed using Mendeley software (Version 1.19.8). The upper limit of the search was limited to December 2020.

### Inclusion and exclusion criteria

Original research articles with ADHD study participants aged ≤18 years, a diagnosis of ADHD according to the Diagnostic and Statistical Manual of Mental Disorders-IV (DSM-IV) or DSM-V criteria, primary research outcomes comprising oculomotor parameters, and articles published in English with a case-control study design were included in this review.

The exclusion criteria were research studies including participants with any other comorbid disorders, research articles in which the participants were on methylphenidate or Ritalin or any other prescribed medication for ADHD during the time of oculomotor testing or on the day of oculomotor testing.

### Article screening

Three authors were involved in the review team. After the initial search in scientific databases, duplicate articles were removed using Mendeley software, and the remaining articles were screened for eligibility. Two authors in the study independently performed title and abstract screening of the included articles. Any disagreement between the two authors in this process was clarified with the third author. Full text of the articles included based on title and abstract screening were assessed for further eligibility as per study inclusion and exclusion criteria. Articles fulfilling the eligibility criteria were reviewed, and data were extracted. The references of all included studies were reviewed for additional relevant publications.

### Data extraction

The extracted data mainly included study title, year of publication, sample size, participants’ age, study design, nature of oculomotor task and outcome measures for the ADHD and control groups. Microsoft Excel (Version 2013) was used for data extraction and management.

### Statistical analysis

Review Manager software (RevMan version 5.4.1) was used for statistical analysis [20]. The analysis included a comparison of various oculomotor parameters between the ADHD and control groups using a Forest plot. This analysis was done only if ≥3 studies reported the same oculomotor parameter as an outcome or a qualitative description of the outcomes was provided. We used random effects as an analytic model because they have better properties in the presence of heterogeneity and are conservative [21]. We used mean difference as an effect measure with a 95% confidence interval. Units of effect measure were entered for the selected variable, and the graph scale range was set accordingly to the selected variable. The heterogeneity of the included studies was tested using *I*^2^ statistics. The value of 0% corresponded to no heterogeneity observed, and a larger heterogeneity value related to increasing heterogeneity [22]. The *I*^2^ value of 25% corresponded to low heterogeneity, 50% corresponded to moderate heterogeneity, and 75% corresponded to high heterogeneity [22].

### Quality assessment

Included studies were assessed for risk of bias by two independent authors using the Modified Newcastle Ottawa scale, a tool for assessing bias in a case-control study [23, 24]. As per this scale, every study received a maximum score of 9, and a score of <5 represented a high risk of bias [25].

## Results

### Identification and selection of the articles for the review

**Fig. 1:**
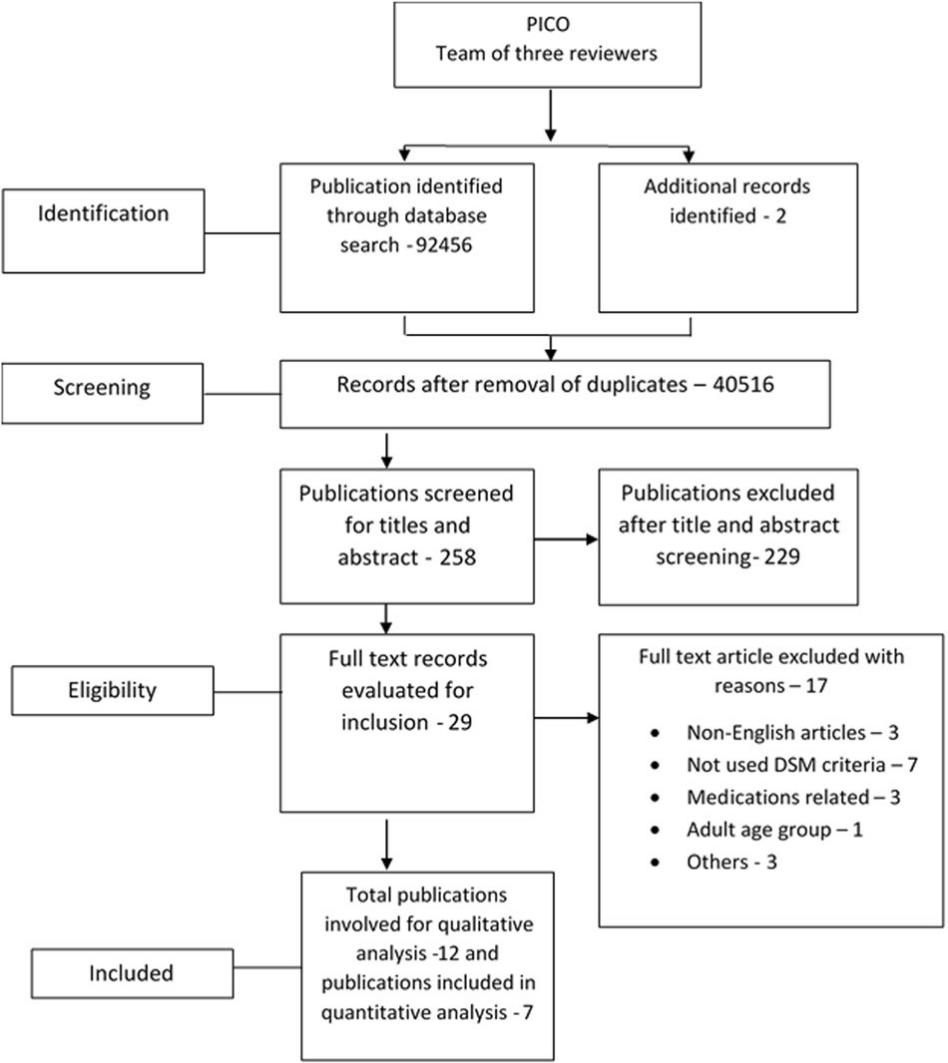
PRISMA flow chart depicting systematic literature search.

Based on our search strategies, a total of 92,456 records were identified from the databases listed in the methodology. The number of articles at various levels of screening is provided in the PRISMA flow chart (Fig. 1). After the abstract and full-text screening, 12 articles met the study criteria and were included in the full-text review.

### Quality of included studies

The risk of bias assessment table for all included studies is given in Table 1. The table shows that there was no high risk of bias for all included studies. Under the exposure subset, for criteria 2, it was seen that no similar questionnaire or tool was used to define cases and controls in six studies denoted in open stars. In the selected subset, criteria 3, three of the studies did not follow proper recruitment and selection of controls.

**Table 1:** Risk of bias assessment of the included studies.

Title	Category								Score
	Selection				Comparability	Exposure			
	1	2	3	4	1	1	2	3	
Castellanos et al. 2000	☆	★	★	★	★	★	★	★	7
Gould et al. 2001	★	★	★	★	★	★	★	★	8
Mostofsky et al. 2001	★	★	★	★	★	★	☆	★	7
Klein et al. 2003	★	★	☆	★	★	★	☆	★	6
Hanisch et al. 2006	☆	★	☆	★	★	★	★	★	6
Van Der Stigchel et al. 2007	★	★	★	☆	★	★	★	★	7
Rommelse et al. 2008	★	★	★	☆	★	★	★	★	7
Loe et al. 2009	★	★	★	★	★	★	☆	★	7
Mahone et al. 2009	★	★	★	★	★	★	★	★	8
Fernandez-Ruiz et al. 2019	★	★	★	★	★	★	☆	★	7
Molina et al. 2020	★	★	☆	★	★	★	☆	★	6
Huang & Chan, 2020	★	★	★	★	★	★	☆	★	7

This table of risk of bias assessment shows that all study has a score >5, suggesting there is no high risk of bias in the included study.

### Study characteristics

Study characteristics in terms of sample size, age range, and outcome measures are given in Table 2. Among the 12 included articles, four studies reported visually guided saccade (VGS) latency, six studies reported antisaccade direction errors, and three studies reported VGS accuracy, MGS accuracy, prosaccade task and intrusive saccade. Two studies reported MGS latency and fixations, and one study reported smooth pursuit measure.

**Table 2:** Characteristics of included studies.

Study article	Study design	Age range in years for cases and controls (y)	Number of children - cases & control	Oculomotor task
Castellanos et al., 2000	Case- Control	ADHD: 6.9 y–10.7 y Controls: 7.9 y –11.3 y	ADHD - 32 Controls- 20	Smooth pursuit, Memory guided saccade
Gould et al., 2001	Case- Control	ADHD and Controls: 7 y–12 y	ADHD - 53 Controls- 44	Fixation task
Mostofsky et al., 2001	Case- Control	ADHD: 7.1y–16.1 y Controls: 7.2y–17.9 y	ADHD - 19 Controls- 25	Memory guided saccade, antisaccade
Klein et al., 2003	Case- Control	ADHD and Controls: 7 y–15 y	ADHD and Controls- 46	Prosaccade, antisaccade
Hanisch et al., 2006	Case- Control	ADHD: 10.7y–13.7 y Controls: 10.7y–12.9 y	ADHD and Controls- 22	Prosaccades, antisaccade, fixations
Van Der Stigchel et al., 2007	Case- Control	ADHD and Controls: 7 y–14 y	ADHD - 22 Controls- 20	Memory guided saccade
Rommelse et al., 2008	Case- Control	ADHD and Controls: 7 y–14 y	ADHD - 22 Controls- 20	Anticipatory and Intrusive saccade
Loe et al., 2009	Case– Control	ADHD and Control: 8 y–13 y	ADHD - 26 Control - 33	Visually guided saccade, antisaccade
Mahone et al., 2009	Case– Control	ADHD and Controls: 8 y–12 y	ADHD and Controls- 60	Memory guided, visually guided saccade & antisaccade
Fernandez-Ruiz et al., 2019	Case– Control	ADHD: 8 y–17 y Controls: 9 y–17 y	ADHD - 22 Controls- 20	Antisaccade
Molina et al., 2020	Case– Control	ADHD: 7.1 y–11.5 y Controls: 6.8 y –11.8 y	ADHD - 21 Controls- 22	Fixations
Huang & Chan, 2020	Case– Control	ADHD: 8 y–9.6 y Controls: 8.8 y–9.1 y	ADHD and Controls- 12	Visually guided saccade, antisaccade

### Prosaccade

Prosaccade is the eye movement made towards a peripheral target from the centre of fixation after a cue presentation [26]. In this task either there will be a delay or an overlap between the time of appearance of the target stimulus and disappearance of the fixation stimulus. The outcomes relevant to prosaccade are reported in terms of prosaccade reaction time, prosaccade accuracy, prosaccade latency and direction errors. Prosaccade accuracy [17] was similar among children with ADHD (7.5 ± 0.8) and without ADHD (7.4 ± 0.6). With respect to prosaccade reaction time, both ADHD (165 ms ± 37 ms) and control groups (174 ms ± 31 ms) showed similar performance [17]. There were no direction errors during the prosaccade task in both groups [27]. Express saccades are referred to as eye movements made with a shorter reaction time (80–130 ms), and express saccades were reduced with increasing age in both groups [27]. During the prosaccade tasks, ADHD children had longer latency (235.6 ms ± 32.4 ms) than the control group (222.7 ms ± 29.3 ms,) but the difference was not statistically significant (*p* = 0.40) [28].

### Memory-guided saccade (MGS)

In MGS, eye movement is made towards the memorised visual target previously seen in the visual periphery [28]. Several studies have evaluated MGS latency, accuracy and anticipatory errors. MGS latency was prolonged among children with ADHD compared to controls [28, 29], and in one study, both groups did not differ with respect to latency [30]. The studies assessing MGS accuracy found that children with ADHD were less accurate than the controls [28, 30, 31]. In the MGS task, the percentage of premature saccades made was higher among children with ADHD than in controls [28]. From the literature, it was seen that ADHD children made significantly greater anticipatory errors than controls in the MGS tasks [28, 30]. Table 3 shows the summary of the mean and standard deviation of various MGS parameters from the studies included in this review.

**Table 3:** Various parameters of memory-guided saccade task.

Task variable	Study	ADHD (Mean ± SD)	Without ADHD (Controls) (Mean ± SD)	*p* value
MGS accuracy (%)	Mostofsky et al., 2001	17.8 ± 9.9	14.9 ± 9.1	0.21
	Mahone et al., 2009	11.5 ± 6.4	12.1 ± 6.0	–
MGS Latency (msec)	Castellanos et al., 2000	365.9 ± 103.8	333.4 ± 76.7	0.33
	Mostofsky et al., 2001	406.3 ± 70.5	345.1 ± 52.7	0.01
Premature saccade (%)	Castellanos et al., 2000	55.2 ± 27.7	32.4 ± 23.2	0.01
Anticipatory errors (%)	Mostofsky et al., 2001	36.4 ± 18.6	22.2 ± 12.0	0.004

### Visually guided saccades (VGS)

**Fig. 2:**
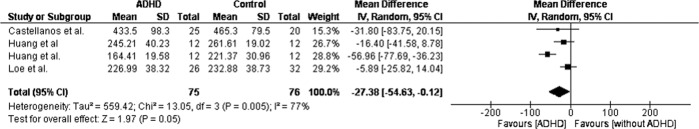
Analysis showing visually guided saccade latency between ADHD and control group. The study by Loe et al. has greater weightage and small CI suggesting statistically significant results. The study by Castellanos et al. has lesser weightage and larger CI stating non-significant results. The overall mean difference is −27.38 with 95% CI [−54.63,−0.12].

VGS are the eye movement made toward a suddenly appearing visual target [32]. Appearance of peripheral target and removal of central fixation occur simultaneously in VGS and the task is to make a saccade to the target. This also referred as reflexive pro-saccade in literature. VGS latency was worse in children with ADHD compared to children without ADHD [29, 31–33]. Girls with ADHD had longer VGS latencies as compared with gender-matched control groups, while such a trend was not noted among boys with ADHD [31]. The forest plot analysis on VGS latency is shown in Fig. 2. The figure shows confidence intervals of three studies [29, 32, 33] passing through the line of no effect, suggesting a statistically non-significant difference, the and *I*^2^ value confirms high heterogeneity. The results are inconsistent due to high heterogeneity and confirm that there is variability in latencies among children with ADHD and without ADHD. Therefore, the evidence for worse VGS latency in the ADHD group compared to age-matched controls is inconclusive.

The studies evaluating VGS accuracy showed that ADHD children had longer accuracy values than children without ADHD, although it was not statistically significant in all studies [29, 32, 33]. Table 4 shows the mean and standard deviation of VGS accuracy and VGS latency parameters.

**Table 4:** VGS accuracy and latency parameters.

VGS variable	Study	ADHD (Mean ± SD)	Without ADHD (Mean ± SD)	*p* value
VGS accuracy %	Castellanos et al., 2000	114.3 ± 68.4	104.6 ± 13.2	0.74
	Huang & Chan, 2020	10.71 ± 9.87	3.06 ± 1.31	<0.05
	Huang & Chan, 2020	7.44 ± 6.82	2.42 ± 1.14	<0.05
VGS latency (msec)	Castellanos et al., 2000	360.9 ± 140.9	210.8 ± 83.7	0.02
	Castellanos et al., 2000	433.5 ± 98.3	465.3 ± 79.5	0.25
	Mahone et al., 2009	238.8 ± 44.1	223 ± 31	0.05
	Loe et al., 2009	226.99 ± 38.32	232.88 ± 38.73	0.566
	Huang & Chan, 2020	164.41 ± 19.58	221.37 ± 30.96	<0.01
	Huang & Chan, 2020	245.21 ± 40.23	261.61 ± 19.02	–

### Antisaccade (AS)

**Fig. 3:**
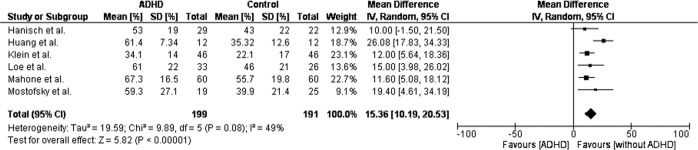
Analysis showing percentage of direction errors made in antisaccade task between ADHD and control group. The figure shows the confidence interval of one study (Hanisch et al.) passing through-line of no effect suggesting statistically non-significant difference, and the *I*^2^ value confirms low heterogeneity. The study by Klein et al. has greater weightage and small CI, suggesting a statistically significant result. The study by Mostofsky et al. has lesser weightage and larger CI, stating non-significant result. The overall mean difference 15.36, with 95% CI [10.19, 20.53]. The overall effect of test *Z* = 5.67 (*P* < 0.001). In analysis, the diamond is on the right side of the graph, favouring the results towards the control group stating that ADHD children made a higher number of direction errors compared to controls.

In AS task, eye movement is made in the direction opposite to the visual stimuli presented [32]. In a study by Huang & Chan, AS latency was shorter among ADHD children than in the control group, and AS accuracy was larger in ADHD children compared to controls. However, these differences between ADHD and controls in AS latency and accuracy were not significant [32]. During the AS task, saccade reaction time was significantly higher among ADHD than in controls [26, 27]. Children with ADHD had a higher percentage of direction errors than those without ADHD [17, 26–28, 31–33]. Table 5 shows the AS parameters extracted from the included studies. The forest plot analysis of AS percentage of direction errors among ADHD children and without ADHD is shown in Fig. 3. Figure 3 shows the confidence interval of one study [17] passing through-line of no effect, suggesting a statistically non-significant difference, and the *I*^2^ value confirms low heterogeneity. In this analysis, the diamond is on the right side of the graph, favouring the results of the control group and depicting that ADHD children made a higher number of direction errors compared to controls.

**Table 5:** Antisaccade parameters.

Antisaccade variable	Study	ADHD (Mean ± SD)	Without ADHD (Mean ± SD)	*P* value
AS Latency (msec)	Huang & Chan, 2020	301.97 ± 62.73	344.60 ± 68.21	–
	Huang & Chan, 2020	330.77 ± 83.55	427.90 ± 48.67	<0.05
AS accuracy (%)	Huang & Chan, 2020	31.52 ± 17.70	30.27 ± 13.34	–
	Huang & Chan, 2020	40.12 ± 22.53	38.52 ± 16.9	–
AS percentage of direction errors (%)	Mostofsky et al., 2001	59.3 ± 27.1	39.9 ± 21.4	<0.01
	Klein et al., 2003 (32)(32)	34.1 ± 14	22.1 ± 17	<0.01
	Hanisch et al., 2006	53 ± 19	43 ± 22	0.16
	Mahone et al., 2009	67.3 ± 16.5	55.7 ± 19.8	0.01
	Loe et al., 2009	61 ± 22	46 ± 21	0.010
	Huang & Chan, 2020	61.40 ± 7.34	35.32 ± 12.6	<0.01
	Huang & Chan, 2020	72.08 ± 8.62	41.47 ± 14.79	<0.01

### Smooth pursuit

There were limited studies on the comparison of pursuits among children with and without ADHD. One publication evaluated smooth pursuit and found no significant difference between the ADHD and control groups [29].

### Fixation

A study evaluated the tendency of visual fixation for 21 s. In this task, children with ADHD made significant eye movement during the 21 s fixation task compared to controls [15].

### Rate of reading

Children with ADHD had significantly more fixations with a low rate of reading, resulting in more time to complete reading tasks, than control children [34].

### Intrusive saccades

Intrusive saccades are defined as inappropriate saccades made during the fixation period. Such saccades were higher among children with ADHD than in the control group [3, 15, 30].

## Discussion

Very few articles explicitly reported the oculomotor deficits present in the ADHD population compared with age-matched controls. We identified 12 case-control studies where various components of oculomotor deficits were compared between children with ADHD and age-matched controls. ADHD children were evaluated in an unmediated state in all studies included in this review. VGS, AS and MGS were commonly reported oculomotor parameters in those studies.

Prosaccade, AS and VGS were assessed based on the response inhibition task. Poor performance in response inhibition tasks is suggestive of frontal lobe deficits [26, 35], and there is hypoactivation of the dorsolateral prefrontal cortex and a decrease in efficiency of the frontostriatal area during response inhibition tasks [26]. Studies assessing prosaccades reported no significant difference between children with and without ADHD. These results suggest that prosaccades are intact among children with ADHD.

The accuracy of MGS was not statistically different between ADHD and control group. Variability in statistical significance of MGS latency reported in two studies could be due to the difference in the delay period used to initiate a MGS after the presentation of a visual target. In one study, a delay period of 1200 msec was used [30], and in another study, a delay period of 4500–5000 msec was used [28], which could have affected the working memory to a greater extent. More anticipatory errors were made during the MGS task. The outcomes of the studies assessing MGS suggest deficits in working memory among individuals with ADHD. MGS is related to working memory tasks. MGS is degraded in ADHD due to the deficiency in visuospatial working memory [28, 36], and it may occur due to intentional inhibition deficits [3]. These abnormalities in the visuospatial working memory could have resulted in decreased accuracy and prolonged latency of MGS [28].

MGS and VGS reflect the function of the cerebellum [37]. VGS had higher accuracy percentage than controls, and the difference was significant in one of the studies [32]. Children with ADHD had longer latency in tasks involving VGS, but the difference was not significant in three studies [29, 32, 33]. The presence of these deficits in ADHD reflects altered oculomotor control, which can occur because of abnormalities in the cerebellum [32, 38, 39]. Longer VGS latencies among the ADHD group have been related to anomalous development in the frontostriatal circuits [40], lesions in the parietal eye field [41] and posterior internal capsule [42]. The difference in VGS latency between children with and without ADHD was highly variable. This could be because of methodological differences regarding fixation duration before the initiation of saccade and the location of the visual stimulus from the centre of fixation.

AS, in terms of accuracy, was not significantly different between children with and without ADHD, but the AS reaction time had a significant difference between both groups. The percentage of direction errors during AS task was higher among the ADHD group than controls. Direction errors attribute to the lack of visual attention in ADHD. The abnormalities in response inhibition contribute to hyperactive, impulsive and off-task behaviour seen in ADHD children [43]. The direction error made by ADHD children during AS tasks is less likely to be improved with ageing because whichever instruction is given to children, they have verbally explained and demonstrated the task [27]. These direction error deficits seen in the AS task are associated with impaired executive function among ADHD children [27].

ADHD children are very slow and will vary in their response to various tasks, which supports an inability or deficit in preparing responses [33]. Additionally, slower saccade search and reaction times are related to the high severity of symptoms [3]. A study comparing ADHD with their non-affected siblings has found that non-affected siblings performed similarly to ADHD children in saccade tasks because the affected siblings share half of their genes with unaffected siblings, and both share and grow in a similar environment [3, 30]. Children with ADHD have problems making effortful inhibition or intentional inhibition response, and they are unaffected by automatic inhibition or implicit form of inhibition [3]. The ADHD children made more intrusive saccades than controls, suggesting that irrelevant saccades are caused by the disorder itself [3]. There is a role of the prefrontal cortex in releasing early response (anticipatory saccade) in saccade tasks [27].

Several studies assessed fixation using the distractor [1, 44, 45], but one of the included studies [15] assessing fixation without the distractor found that large saccades were made during fixation. ADHD children have difficulty inhibiting prepotent reactions and difficulty maintaining fixation in the absence of a distractor due to frontostriatal dysfunction [46]. The involvement of the frontal eye field delays the release of visual attention and fixation [27]. In poor reading performance, it is related to working memory deficits [34]. This study data stated that children with ADHD have difficulty maintaining fixations, and deficient eye movement pattern was associated with poor reading skills.

The cerebellar dysfunction and delayed maturation in basal ganglion contribute to impaired spatial attention and voluntary eye movement in ADHD children. They also reduce hand-eye coordination and visual-spatial recognition among ADHD patients [32]. The presence of deficits in AS, MGS and VGS is associated with abnormalities within the prefrontal cortex and basal ganglia [28].

This review witnessed the extent of statistical heterogeneity in the studies reporting VGS latency. The VGS latency was associated with a significantly high *I*^2^ value, suggesting the variability across the studies. In the AS task, the percentage of direction errors made was more in ADHD children. Overall, children with ADHD exhibited less precision in making eye movements and required more time to complete the oculomotor task compared to children without ADHD. Although individual studies reported that ADHD children had poorer performance compared to children without ADHD, there is a lack of strong evidence to confirm the presence of oculomotor deficits because of the heterogeneity among the studies. In addition, there is a lack of studies on the assessment of smooth pursuit among ADHD children compared with controls. There is a need for studies with standardised and uniform experimental design to substantiate the oculomotor deficits among children with ADHD.

Supplemental Material is available at nature.com/eye

## Supplementary information

Supplemental Material
