# Supplementary material for: Oculomotor deficits in attention deficit hyperactivity disorder: a systematic review and meta-analysis
Source: Eye (Lond). 2022 Oct 24;37(10):1975–81. doi: 10.1038/s41433-022-02284-z (PMC10333290; doi:10.1038/s41433-022-02284-z)
Supplement: Supplementary file 1 — Supplemental Material [file 41433_2022_2284_MOESM1_ESM.docx]

**Supplementary section**

**Search string used through various electronic database**

| **Search string**  **PubMed** | **Retrieved Results** |
| --- | --- |
| (("attention deficit disorder with hyperactivity"[MeSH Terms] OR ("attention"[All Fields] AND "deficit"[All Fields] AND "disorder"[All Fields] AND "hyperactivity"[All Fields]) OR "attention deficit disorder with hyperactivity"[All Fields] OR ("attention"[All Fields] AND "deficit"[All Fields] AND "hyperactivity"[All Fields] AND "disorder"[All Fields]) OR "attention deficit hyperactivity disorder"[All Fields] OR "attention deficit disorder with hyperactivity"[MeSH Terms] OR "attention deficit hyperactivity disorder"[Title/Abstract] OR ("attention deficit disorder with hyperactivity"[MeSH Terms] OR ("attention"[All Fields] AND "deficit"[All Fields] AND "disorder"[All Fields] AND "hyperactivity"[All Fields]) OR "attention deficit disorder with hyperactivity"[All Fields] OR "ADHD"[All Fields]) OR "attention deficit disorder with hyperactivity"[MeSH Terms] OR "ADHD"[Title/Abstract]) AND ("saccade s"[All Fields] OR "saccaded"[All Fields] OR "saccades"[MeSH Terms] OR "saccades"[All Fields] OR "saccade"[All Fields] OR "saccadic"[All Fields] OR "saccadically"[All Fields] OR "saccading"[All Fields])) OR "saccades"[MeSH Terms] OR "saccade"[Title/Abstract] OR ("saccade s"[All Fields] OR "saccaded"[All Fields] OR "saccades"[MeSH Terms] OR "saccades"[All Fields] OR "saccade"[All Fields] OR "saccadic"[All Fields] OR "saccadically"[All Fields] OR "saccading"[All Fields]) OR "saccades"[Title/Abstract] OR "saccades"[MeSH Terms] OR ("saccade s"[All Fields] OR "saccaded"[All Fields] OR "saccades"[MeSH Terms] OR "saccades"[All Fields] OR "saccade"[All Fields] OR "saccadic"[All Fields] OR "saccadically"[All Fields] OR "saccading"[All Fields]) OR "saccadic"[Title/Abstract] OR ("saccades"[MeSH Terms] OR "saccades"[All Fields] OR ("saccadic"[All Fields] AND "eye"[All Fields] AND "movement"[All Fields]) OR "saccadic eye movement"[All Fields]) OR "saccadic eye movement"[Title/Abstract] OR "saccades"[MeSH Terms] OR (("saccade s"[All Fields] OR "saccaded"[All Fields] OR "saccades"[MeSH Terms] OR "saccades"[All Fields] OR "saccade"[All Fields] OR "saccadic"[All Fields] OR "saccadically"[All Fields] OR "saccading"[All Fields]) AND ("eye"[MeSH Terms] OR "eye"[All Fields])) OR "saccadic eye"[Title/Abstract] OR (("saccade s"[All Fields] OR "saccaded"[All Fields] OR "saccades"[MeSH Terms] OR "saccades"[All Fields] OR "saccade"[All Fields] OR "saccadic"[All Fields] OR "saccadically"[All Fields] OR "saccading"[All Fields]) AND "eye"[MeSH Terms]) OR ("pursuit"[All Fields] OR "pursuits"[All Fields]) OR "pursuit"[Title/Abstract] OR ("pursuit"[All Fields] OR "pursuits"[All Fields]) OR "pursuits"[Title/Abstract] OR ("pursuit, smooth"[MeSH Terms] OR ("pursuit"[All Fields] AND "smooth"[All Fields]) OR "smooth pursuit"[All Fields] OR ("smooth"[All Fields] AND "pursuit"[All Fields])) OR "smooth pursuit"[Title/Abstract] OR "pursuit, smooth"[MeSH Terms] OR ("pursuit, smooth"[MeSH Terms] OR ("pursuit"[All Fields] AND "smooth"[All Fields]) OR "smooth pursuit"[All Fields] OR ("smooth"[All Fields] AND "pursuits"[All Fields]) OR "smooth pursuits"[All Fields]) OR "smooth pursuits"[Title/Abstract] OR "pursuit, smooth"[MeSH Terms] OR (("smooth"[All Fields] OR "smoothe"[All Fields] OR "smoothed"[All Fields] OR "smoothes"[All Fields] OR "smoothing"[All Fields] OR "smoothings"[All Fields] OR "smoothness"[All Fields] OR "smooths"[All Fields]) AND ("eye movements"[MeSH Terms] OR ("eye"[All Fields] AND "movements"[All Fields]) OR "eye movements"[All Fields] OR ("eye"[All Fields] AND "movement"[All Fields]) OR "eye movement"[All Fields])) OR "smooth eye movement"[Title/Abstract] OR (("smooth"[All Fields] OR "smoothe"[All Fields] OR "smoothed"[All Fields] OR "smoothes"[All Fields] OR "smoothing"[All Fields] OR "smoothings"[All Fields] OR "smoothness"[All Fields] OR "smooths"[All Fields]) AND "eye movements"[MeSH Terms]) OR (("oculomotor"[All Fields] OR "oculomotoric"[All Fields]) AND ("abnormal"[All Fields] OR "abnormalities"[MeSH Subheading] OR "abnormalities"[All Fields] OR "congenital abnormalities"[MeSH Terms] OR ("congenital"[All Fields] AND "abnormalities"[All Fields]) OR "congenital abnormalities"[All Fields] OR "abnormality"[All Fields] OR "abnormally"[All Fields] OR "abnormals"[All Fields] OR "abnormities"[All Fields] OR "abnormity"[All Fields])) OR "oculomotor abnormalities"[Title/Abstract] OR (("oculomotor"[All Fields] OR "oculomotoric"[All Fields]) AND "congenital abnormalities"[MeSH Terms]) OR (("oculomotor"[All Fields] OR "oculomotoric"[All Fields]) AND ("deficit"[All Fields] OR "deficits"[All Fields])) OR "oculomotor deficit"[Title/Abstract] OR (("oculomotor"[All Fields] OR "oculomotoric"[All Fields]) AND ("abnormalities"[MeSH Subheading] OR "abnormalities"[All Fields] OR "anomalies"[All Fields] OR "anomalie"[All Fields] OR "anomaly"[All Fields])) OR "oculomotor anomalies"[Title/Abstract] OR (("oculomotor"[All Fields] OR "oculomotoric"[All Fields]) AND ("deficit"[All Fields] OR "deficits"[All Fields])) OR "oculomotor deficits"[Title/Abstract] OR (("oculomotor"[All Fields] OR "oculomotoric"[All Fields]) AND ("problem"[All Fields] OR "problem s"[All Fields] OR "problems"[All Fields])) OR "oculomotor problem"[Title/Abstract] OR (("oculomotor"[All Fields] OR "oculomotoric"[All Fields]) AND ("problem"[All Fields] OR "problem s"[All Fields] OR "problems"[All Fields])) OR "oculomotor problems"[Title/Abstract] OR (("oculomotor"[All Fields] OR "oculomotoric"[All Fields]) AND "defect"[Title/Abstract]) OR (("oculomotor"[All Fields] OR "oculomotoric"[All Fields]) AND ("disease"[MeSH Terms] OR "disease"[All Fields] OR "disorder"[All Fields] OR "disorders"[All Fields] OR "disorder s"[All Fields] OR "disordes"[All Fields])) OR "oculomotor disorder"[Title/Abstract] OR (("oculomotor"[All Fields] OR "oculomotoric"[All Fields]) AND "disease"[MeSH Terms]) | 40173 |
| (("attention deficit disorder with hyperactivity"[MeSH Terms] OR ("attention"[All Fields] AND "deficit"[All Fields] AND "disorder"[All Fields] AND "hyperactivity"[All Fields]) OR "attention deficit disorder with hyperactivity"[All Fields] OR ("attention"[All Fields] AND "deficit"[All Fields] AND "hyperactivity"[All Fields] AND "disorder"[All Fields]) OR "attention deficit hyperactivity disorder"[All Fields] OR "attention deficit disorder with hyperactivity"[MeSH Terms] OR "attention deficit hyperactivity disorder"[Title/Abstract] OR ("attention deficit disorder with hyperactivity"[MeSH Terms] OR ("attention"[All Fields] AND "deficit"[All Fields] AND "disorder"[All Fields] AND "hyperactivity"[All Fields]) OR "attention deficit disorder with hyperactivity"[All Fields] OR "ADHD"[All Fields]) OR "attention deficit disorder with hyperactivity"[MeSH Terms] OR "ADHD"[Title/Abstract]) AND ("saccade s"[All Fields] OR "saccaded"[All Fields] OR "saccades"[MeSH Terms] OR "saccades"[All Fields] OR "saccade"[All Fields] OR "saccadic"[All Fields] OR "saccadically"[All Fields] OR "saccading"[All Fields])) OR "saccades"[MeSH Terms] OR "saccade"[Title/Abstract] OR ("saccade s"[All Fields] OR "saccaded"[All Fields] OR "saccades"[MeSH Terms] OR "saccades"[All Fields] OR "saccade"[All Fields] OR "saccadic"[All Fields] OR "saccadically"[All Fields] OR "saccading"[All Fields]) OR "saccades"[Title/Abstract] OR "saccades"[MeSH Terms] OR ("saccade s"[All Fields] OR "saccaded"[All Fields] OR "saccades"[MeSH Terms] OR "saccades"[All Fields] OR "saccade"[All Fields] OR "saccadic"[All Fields] OR "saccadically"[All Fields] OR "saccading"[All Fields]) OR "saccadic"[Title/Abstract] OR ("saccades"[MeSH Terms] OR "saccades"[All Fields] OR ("saccadic"[All Fields] AND "eye"[All Fields] AND "movement"[All Fields]) OR "saccadic eye movement"[All Fields]) OR "saccadic eye movement"[Title/Abstract] OR "saccades"[MeSH Terms] OR (("saccade s"[All Fields] OR "saccaded"[All Fields] OR "saccades"[MeSH Terms] OR "saccades"[All Fields] OR "saccade"[All Fields] OR "saccadic"[All Fields] OR "saccadically"[All Fields] OR "saccading"[All Fields]) AND ("eye"[MeSH Terms] OR "eye"[All Fields])) OR "saccadic eye"[Title/Abstract] OR (("saccade s"[All Fields] OR "saccaded"[All Fields] OR "saccades"[MeSH Terms] OR "saccades"[All Fields] OR "saccade"[All Fields] OR "saccadic"[All Fields] OR "saccadically"[All Fields] OR "saccading"[All Fields]) AND "eye"[MeSH Terms]) OR ("pursuit"[All Fields] OR "pursuits"[All Fields]) OR "pursuit"[Title/Abstract] OR ("pursuit"[All Fields] OR "pursuits"[All Fields]) OR "pursuits"[Title/Abstract] OR ("pursuit, smooth"[MeSH Terms] OR ("pursuit"[All Fields] AND "smooth"[All Fields]) OR "smooth pursuit"[All Fields] OR ("smooth"[All Fields] AND "pursuit"[All Fields])) OR "smooth pursuit"[Title/Abstract] OR "pursuit, smooth"[MeSH Terms] OR ("pursuit, smooth"[MeSH Terms] OR ("pursuit"[All Fields] AND "smooth"[All Fields]) OR "smooth pursuit"[All Fields] OR ("smooth"[All Fields] AND "pursuits"[All Fields]) OR "smooth pursuits"[All Fields]) OR "smooth pursuits"[Title/Abstract] OR "pursuit, smooth"[MeSH Terms] OR (("smooth"[All Fields] OR "smoothe"[All Fields] OR "smoothed"[All Fields] OR "smoothes"[All Fields] OR "smoothing"[All Fields] OR "smoothings"[All Fields] OR "smoothness"[All Fields] OR "smooths"[All Fields]) AND ("eye movements"[MeSH Terms] OR ("eye"[All Fields] AND "movements"[All Fields]) OR "eye movements"[All Fields] OR ("eye"[All Fields] AND "movement"[All Fields]) OR "eye movement"[All Fields])) OR "smooth eye movement"[Title/Abstract] OR (("smooth"[All Fields] OR "smoothe"[All Fields] OR "smoothed"[All Fields] OR "smoothes"[All Fields] OR "smoothing"[All Fields] OR "smoothings"[All Fields] OR "smoothness"[All Fields] OR "smooths"[All Fields]) AND "eye movements"[MeSH Terms]) OR (("oculomotor"[All Fields] OR "oculomotoric"[All Fields]) AND ("abnormal"[All Fields] OR "abnormalities"[MeSH Subheading] OR "abnormalities"[All Fields] OR "congenital abnormalities"[MeSH Terms] OR ("congenital"[All Fields] AND "abnormalities"[All Fields]) OR "congenital abnormalities"[All Fields] OR "abnormality"[All Fields] OR "abnormally"[All Fields] OR "abnormals"[All Fields] OR "abnormities"[All Fields] OR "abnormity"[All Fields])) OR "oculomotor abnormalities"[Title/Abstract] OR (("oculomotor"[All Fields] OR "oculomotoric"[All Fields]) AND "congenital abnormalities"[MeSH Terms]) OR (("oculomotor"[All Fields] OR "oculomotoric"[All Fields]) AND ("deficit"[All Fields] OR "deficits"[All Fields])) OR "oculomotor deficit"[Title/Abstract] OR (("oculomotor"[All Fields] OR "oculomotoric"[All Fields]) AND ("abnormalities"[MeSH Subheading] OR "abnormalities"[All Fields] OR "anomalies"[All Fields] OR "anomalie"[All Fields] OR "anomaly"[All Fields])) OR "oculomotor anomalies"[Title/Abstract] OR (("oculomotor"[All Fields] OR "oculomotoric"[All Fields]) AND ("deficit"[All Fields] OR "deficits"[All Fields])) OR "oculomotor deficits"[Title/Abstract] OR (("oculomotor"[All Fields] OR "oculomotoric"[All Fields]) AND ("problem"[All Fields] OR "problem s"[All Fields] OR "problems"[All Fields])) OR "oculomotor problem"[Title/Abstract] OR (("oculomotor"[All Fields] OR "oculomotoric"[All Fields]) AND ("problem"[All Fields] OR "problem s"[All Fields] OR "problems"[All Fields])) OR "oculomotor problems"[Title/Abstract] OR (("oculomotor"[All Fields] OR "oculomotoric"[All Fields]) AND "defect"[Title/Abstract]) OR (("oculomotor"[All Fields] OR "oculomotoric"[All Fields]) AND ("disease"[MeSH Terms] OR "disease"[All Fields] OR "disorder"[All Fields] OR "disorders"[All Fields] OR "disorder s"[All Fields] OR "disordes"[All Fields])) OR "oculomotor disorder"[Title/Abstract] OR (("oculomotor"[All Fields] OR "oculomotoric"[All Fields]) AND "disease"[MeSH Terms]) | 6558 |
| (("oculomotor deficit"[Title/Abstract] OR "oculomotor problem"[Title/Abstract] OR "oculomotor abnormalities"[Title/Abstract] OR "oculomotor anomalies"[Title/Abstract] OR "oculomotor problems"[Title/Abstract] OR "oculomotor defects"[Title/Abstract] OR "saccade"[Title/Abstract] OR "saccades"[Title/Abstract] OR "saccadic"[Title/Abstract] OR "saccadic eye movement"[Title/Abstract] OR "pursuit"[Title/Abstract] OR "smooth pursuit"[Title/Abstract] OR "smooth pursuits"[Title/Abstract] OR "visual fixation"[Title/Abstract] OR "pursuits"[Title/Abstract] OR ((("Oculomotor"[All Fields] OR "oculomotoric"[All Fields]) AND ("Deficit"[All Fields] OR "deficits"[All Fields])) OR (("Oculomotor"[All Fields] OR "oculomotoric"[All Fields]) AND ("problem"[All Fields] OR "problem s"[All Fields] OR "problems"[All Fields])) OR (("Oculomotor"[All Fields] OR "oculomotoric"[All Fields]) AND ("abnormal"[All Fields] OR "abnormalities"[MeSH Subheading] OR "abnormalities"[All Fields] OR "congenital abnormalities"[MeSH Terms] OR ("congenital"[All Fields] AND "abnormalities"[All Fields]) OR "congenital abnormalities"[All Fields] OR "abnormality"[All Fields] OR "abnormally"[All Fields] OR "abnormals"[All Fields] OR "abnormities"[All Fields] OR "abnormity"[All Fields])) OR (("Oculomotor"[All Fields] OR "oculomotoric"[All Fields]) AND ("abnormalities"[MeSH Subheading] OR "abnormalities"[All Fields] OR "anomalies"[All Fields] OR "anomalie"[All Fields] OR "anomaly"[All Fields])) OR (("Oculomotor"[All Fields] OR "oculomotoric"[All Fields]) AND ("problem"[All Fields] OR "problem s"[All Fields] OR "problems"[All Fields])) OR (("Oculomotor"[All Fields] OR "oculomotoric"[All Fields]) AND ("abnormalities"[MeSH Subheading] OR "abnormalities"[All Fields] OR "defects"[All Fields] OR "defect"[All Fields] OR "defect s"[All Fields] OR "defected"[All Fields] OR "defective"[All Fields] OR "defectively"[All Fields] OR "defectives"[All Fields])) OR ("saccade s"[All Fields] OR "saccaded"[All Fields] OR "saccades"[MeSH Terms] OR "saccades"[All Fields] OR "saccade"[All Fields] OR "saccadic"[All Fields] OR "saccadically"[All Fields] OR "saccading"[All Fields]) OR ("saccade s"[All Fields] OR "saccaded"[All Fields] OR "saccades"[MeSH Terms] OR "saccades"[All Fields] OR "saccade"[All Fields] OR "saccadic"[All Fields] OR "saccadically"[All Fields] OR "saccading"[All Fields]) OR ("saccade s"[All Fields] OR "saccaded"[All Fields] OR "saccades"[MeSH Terms] OR "saccades"[All Fields] OR "saccade"[All Fields] OR "saccadic"[All Fields] OR "saccadically"[All Fields] OR "saccading"[All Fields]) OR ("saccades"[MeSH Terms] OR "saccades"[All Fields] OR ("saccadic"[All Fields] AND "eye"[All Fields] AND "movement"[All Fields]) OR "saccadic eye movement"[All Fields]) OR ("pursuit"[All Fields] OR "pursuits"[All Fields]) OR ("pursuit, smooth"[MeSH Terms] OR ("pursuit"[All Fields] AND "smooth"[All Fields]) OR "smooth pursuit"[All Fields] OR ("smooth"[All Fields] AND "pursuit"[All Fields])) OR ("pursuit, smooth"[MeSH Terms] OR ("pursuit"[All Fields] AND "smooth"[All Fields]) OR "smooth pursuit"[All Fields] OR ("smooth"[All Fields] AND "pursuits"[All Fields]) OR "smooth pursuits"[All Fields]) OR ("fixation, ocular"[MeSH Terms] OR ("fixation"[All Fields] AND "ocular"[All Fields]) OR "ocular fixation"[All Fields] OR ("visual"[All Fields] AND "fixation"[All Fields]) OR "visual fixation"[All Fields]) OR ("pursuit"[All Fields] OR "pursuits"[All Fields]))) AND ("attention deficit disorder with hyperactivity"[MeSH Terms] OR ("attention"[All Fields] AND "Deficit"[All Fields] AND "disorder"[All Fields] AND "hyperactivity"[All Fields]) OR "attention deficit disorder with hyperactivity"[All Fields] OR ("attention"[All Fields] AND "Deficit"[All Fields] AND "hyperactivity"[All Fields] AND "disorder"[All Fields]) OR "attention deficit hyperactivity disorder"[All Fields])) OR ("attention deficit disorder with hyperactivity"[MeSH Terms] OR ("attention"[All Fields] AND "Deficit"[All Fields] AND "disorder"[All Fields] AND "hyperactivity"[All Fields]) OR "attention deficit disorder with hyperactivity"[All Fields] OR "ADHD"[All Fields]) OR "attention deficit hyperactivity disorder"[Title/Abstract] OR "ADHD"[Title/Abstract] | 11908 |
| (("oculomotor deficit"[Title/Abstract] OR "oculomotor problem"[Title/Abstract] OR "oculomotor abnormalities"[Title/Abstract] OR "oculomotor anomalies"[Title/Abstract] OR "oculomotor problems"[Title/Abstract] OR "oculomotor defects"[Title/Abstract] OR "saccade"[Title/Abstract] OR "saccades"[Title/Abstract] OR "saccadic"[Title/Abstract] OR "saccadic eye movement"[Title/Abstract] OR "pursuit"[Title/Abstract] OR "smooth pursuit"[Title/Abstract] OR "smooth pursuits"[Title/Abstract] OR "visual fixation"[Title/Abstract] OR "pursuits"[Title/Abstract] OR ((("Oculomotor"[All Fields] OR "oculomotoric"[All Fields]) AND ("Deficit"[All Fields] OR "deficits"[All Fields])) OR (("Oculomotor"[All Fields] OR "oculomotoric"[All Fields]) AND ("problem"[All Fields] OR "problem s"[All Fields] OR "problems"[All Fields])) OR (("Oculomotor"[All Fields] OR "oculomotoric"[All Fields]) AND ("abnormal"[All Fields] OR "abnormalities"[MeSH Subheading] OR "abnormalities"[All Fields] OR "congenital abnormalities"[MeSH Terms] OR ("congenital"[All Fields] AND "abnormalities"[All Fields]) OR "congenital abnormalities"[All Fields] OR "abnormality"[All Fields] OR "abnormally"[All Fields] OR "abnormals"[All Fields] OR "abnormities"[All Fields] OR "abnormity"[All Fields])) OR (("Oculomotor"[All Fields] OR "oculomotoric"[All Fields]) AND ("abnormalities"[MeSH Subheading] OR "abnormalities"[All Fields] OR "anomalies"[All Fields] OR "anomalie"[All Fields] OR "anomaly"[All Fields])) OR (("Oculomotor"[All Fields] OR "oculomotoric"[All Fields]) AND ("problem"[All Fields] OR "problem s"[All Fields] OR "problems"[All Fields])) OR (("Oculomotor"[All Fields] OR "oculomotoric"[All Fields]) AND ("abnormalities"[MeSH Subheading] OR "abnormalities"[All Fields] OR "defects"[All Fields] OR "defect"[All Fields] OR "defect s"[All Fields] OR "defected"[All Fields] OR "defective"[All Fields] OR "defectively"[All Fields] OR "defectives"[All Fields])) OR ("saccade s"[All Fields] OR "saccaded"[All Fields] OR "saccades"[MeSH Terms] OR "saccades"[All Fields] OR "saccade"[All Fields] OR "saccadic"[All Fields] OR "saccadically"[All Fields] OR "saccading"[All Fields]) OR ("saccade s"[All Fields] OR "saccaded"[All Fields] OR "saccades"[MeSH Terms] OR "saccades"[All Fields] OR "saccade"[All Fields] OR "saccadic"[All Fields] OR "saccadically"[All Fields] OR "saccading"[All Fields]) OR ("saccade s"[All Fields] OR "saccaded"[All Fields] OR "saccades"[MeSH Terms] OR "saccades"[All Fields] OR "saccade"[All Fields] OR "saccadic"[All Fields] OR "saccadically"[All Fields] OR "saccading"[All Fields]) OR ("saccades"[MeSH Terms] OR "saccades"[All Fields] OR ("saccadic"[All Fields] AND "eye"[All Fields] AND "movement"[All Fields]) OR "saccadic eye movement"[All Fields]) OR ("pursuit"[All Fields] OR "pursuits"[All Fields]) OR ("pursuit, smooth"[MeSH Terms] OR ("pursuit"[All Fields] AND "smooth"[All Fields]) OR "smooth pursuit"[All Fields] OR ("smooth"[All Fields] AND "pursuit"[All Fields])) OR ("pursuit, smooth"[MeSH Terms] OR ("pursuit"[All Fields] AND "smooth"[All Fields]) OR "smooth pursuit"[All Fields] OR ("smooth"[All Fields] AND "pursuits"[All Fields]) OR "smooth pursuits"[All Fields]) OR ("fixation, ocular"[MeSH Terms] OR ("fixation"[All Fields] AND "ocular"[All Fields]) OR "ocular fixation"[All Fields] OR ("visual"[All Fields] AND "fixation"[All Fields]) OR "visual fixation"[All Fields]) OR ("pursuit"[All Fields] OR "pursuits"[All Fields]))) AND ("attention deficit disorder with hyperactivity"[MeSH Terms] OR ("attention"[All Fields] AND "Deficit"[All Fields] AND "disorder"[All Fields] AND "hyperactivity"[All Fields]) OR "attention deficit disorder with hyperactivity"[All Fields] OR ("attention"[All Fields] AND "Deficit"[All Fields] AND "hyperactivity"[All Fields] AND "disorder"[All Fields]) OR "attention deficit hyperactivity disorder"[All Fields])) OR ("attention deficit disorder with hyperactivity"[MeSH Terms] OR ("attention"[All Fields] AND "Deficit"[All Fields] AND "disorder"[All Fields] AND "hyperactivity"[All Fields]) OR "attention deficit disorder with hyperactivity"[All Fields] OR "ADHD"[All Fields]) OR "attention deficit hyperactivity disorder"[Title/Abstract] OR "ADHD"[Title/Abstract] | 6146 |
| (("attention deficit disorder with hyperactivity"[MeSH Terms] OR ("attention"[All Fields] AND "deficit"[All Fields] AND "disorder"[All Fields] AND "hyperactivity"[All Fields]) OR "attention deficit disorder with hyperactivity"[All Fields] OR ("attention"[All Fields] AND "deficit"[All Fields] AND "hyperactivity"[All Fields] AND "disorder"[All Fields]) OR "attention deficit hyperactivity disorder"[All Fields] OR "attention deficit disorder with hyperactivity"[MeSH Terms] OR ("attention deficit disorder with hyperactivity"[MeSH Terms] OR ("attention"[All Fields] AND "deficit"[All Fields] AND "disorder"[All Fields] AND "hyperactivity"[All Fields]) OR "attention deficit disorder with hyperactivity"[All Fields] OR "adhd"[All Fields]) OR "attention deficit disorder with hyperactivity"[MeSH Terms]) AND ("saccade s"[All Fields] OR "saccaded"[All Fields] OR "saccades"[MeSH Terms] OR "saccades"[All Fields] OR "saccade"[All Fields] OR "saccadic"[All Fields] OR "saccadically"[All Fields] OR "saccading"[All Fields])) OR "saccades"[MeSH Terms] OR ("pursuit"[All Fields] OR "pursuits"[All Fields]) OR ("pursuit, smooth"[MeSH Terms] OR ("pursuit"[All Fields] AND "smooth"[All Fields]) OR "smooth pursuit"[All Fields] OR ("smooth"[All Fields] AND "pursuit"[All Fields])) OR "pursuit, smooth"[MeSH Terms] OR ("oculomotor"[All Fields] OR "oculomotoric"[All Fields]) OR ("fixate"[All Fields] OR "fixated"[All Fields] OR "fixates"[All Fields] OR "fixating"[All Fields] OR "fixation"[All Fields] OR "fixational"[All Fields] OR "fixations"[All Fields] OR "fixator"[All Fields] OR "fixator s"[All Fields] OR "fixators"[All Fields]) | 6444 |
| **PMC** |  |
| (("saccades"[MeSH Terms] OR "saccades"[All Fields] OR "saccade"[All Fields]) OR oculomotor[All Fields] OR pursuit[All Fields] OR fixation[All Fields]) AND (("attention deficit disorder with hyperactivity"[MeSH Terms] OR ("attention"[All Fields] AND "deficit"[All Fields] AND "disorder"[All Fields] AND "hyperactivity"[All Fields]) OR "attention deficit disorder with hyperactivity"[All Fields] OR ("attention"[All Fields] AND "deficit"[All Fields] AND "hyperactivity"[All Fields] AND "disorder"[All Fields]) OR "attention deficit hyperactivity disorder"[All Fields]) OR ("attention deficit disorder with hyperactivity"[MeSH Terms] OR ("attention"[All Fields] AND "deficit"[All Fields] AND "disorder"[All Fields] AND "hyperactivity"[All Fields]) OR "attention deficit disorder with hyperactivity"[All Fields] OR "adhd"[All Fields])) | 7834 |
| (("attention deficit disorder with hyperactivity"[MeSH Terms] OR ("attention"[All Fields] AND "deficit"[All Fields] AND "disorder"[All Fields] AND "hyperactivity"[All Fields]) OR "attention deficit disorder with hyperactivity"[All Fields] OR ("attention"[All Fields] AND "deficit"[All Fields] AND "hyperactivity"[All Fields] AND "disorder"[All Fields]) OR "attention deficit hyperactivity disorder"[All Fields]) OR ("attention deficit disorder with hyperactivity"[MeSH Terms] OR ("attention"[All Fields] AND "deficit"[All Fields] AND "disorder"[All Fields] AND "hyperactivity"[All Fields]) OR "attention deficit disorder with hyperactivity"[All Fields] OR "adhd"[All Fields])) AND (("saccades"[MeSH Terms] OR "saccades"[All Fields] OR "saccade"[All Fields]) OR oculomotor[All Fields] OR pursuit[All Fields] OR fixation[All Fields]) | 7841 |
| (((("saccades"[MeSH Terms] OR "saccades"[All Fields] OR "saccade"[All Fields]) OR ("saccades"[MeSH Terms] OR "saccades"[All Fields]) OR ("saccades"[MeSH Terms] OR "saccades"[All Fields] OR ("saccadic"[All Fields] AND "eye"[All Fields] AND "movement"[All Fields]) OR "saccadic eye movement"[All Fields]) OR saccadic[All Fields]) OR (pursuit[All Fields] OR ("pursuit, smooth"[MeSH Terms] OR ("pursuit"[All Fields] AND "smooth"[All Fields]) OR "smooth pursuit"[All Fields] OR ("smooth"[All Fields] AND "pursuit"[All Fields])) OR pursuits[All Fields] OR ("pursuit, smooth"[MeSH Terms] OR ("pursuit"[All Fields] AND "smooth"[All Fields]) OR "smooth pursuit"[All Fields] OR ("smooth"[All Fields] AND "pursuits"[All Fields]) OR "smooth pursuits"[All Fields]))) OR (oculomotor[All Fields] OR (oculomotor[All Fields] AND ("abnormalities"[Subheading] OR "abnormalities"[All Fields] OR "congenital abnormalities"[MeSH Terms] OR ("congenital"[All Fields] AND "abnormalities"[All Fields]) OR "congenital abnormalities"[All Fields])) OR (oculomotor[All Fields] AND ("abnormalities"[Subheading] OR "abnormalities"[All Fields] OR "anomalies"[All Fields])))) AND (("attention deficit disorder with hyperactivity"[MeSH Terms] OR ("attention"[All Fields] AND "deficit"[All Fields] AND "disorder"[All Fields] AND "hyperactivity"[All Fields]) OR "attention deficit disorder with hyperactivity"[All Fields] OR ("attention"[All Fields] AND "deficit"[All Fields] AND "hyperactivity"[All Fields] AND "disorder"[All Fields]) OR "attention deficit hyperactivity disorder"[All Fields]) OR ("attention deficit disorder with hyperactivity"[MeSH Terms] OR ("attention"[All Fields] AND "deficit"[All Fields] AND "disorder"[All Fields] AND "hyperactivity"[All Fields]) OR "attention deficit disorder with hyperactivity"[All Fields] OR "adhd"[All Fields])) | 4362 |
| ((((("saccades"[MeSH Terms] OR "saccades"[All Fields] OR "saccade"[All Fields]) OR pursuit[All Fields]) OR fixation[All Fields]) OR oculomotor[All Fields]) AND ("attention deficit disorder with hyperactivity"[MeSH Terms] OR ("attention"[All Fields] AND "deficit"[All Fields] AND "disorder"[All Fields] AND "hyperactivity"[All Fields]) OR "attention deficit disorder with hyperactivity"[All Fields] OR ("attention"[All Fields] AND "deficit"[All Fields] AND "hyperactivity"[All Fields] AND "disorder"[All Fields]) OR "attention deficit hyperactivity disorder"[All Fields])) AND ("attention deficit disorder with hyperactivity"[MeSH Terms] OR ("attention"[All Fields] AND "deficit"[All Fields] AND "disorder"[All Fields] AND "hyperactivity"[All Fields]) OR "attention deficit disorder with hyperactivity"[All Fields] OR "adhd"[All Fields]) | 6308 |
| (("attention deficit disorder with hyperactivity"[MeSH Terms] OR ("attention"[All Fields] AND "deficit"[All Fields] AND "disorder"[All Fields] AND "hyperactivity"[All Fields]) OR "attention deficit disorder with hyperactivity"[All Fields] OR ("attention"[All Fields] AND "deficit"[All Fields] AND "hyperactivity"[All Fields] AND "disorder"[All Fields]) OR "attention deficit hyperactivity disorder"[All Fields]) OR ("attention deficit disorder with hyperactivity"[MeSH Terms] OR ("attention"[All Fields] AND "deficit"[All Fields] AND "disorder"[All Fields] AND "hyperactivity"[All Fields]) OR "attention deficit disorder with hyperactivity"[All Fields] OR "adhd"[All Fields])) AND (("saccades"[MeSH Terms] OR "saccades"[All Fields] OR "saccade"[All Fields]) OR saccadic[All Fields]) | 1956 |
| (("attention deficit disorder with hyperactivity"[MeSH Terms] OR ("attention"[All Fields] AND "deficit"[All Fields] AND "disorder"[All Fields] AND "hyperactivity"[All Fields]) OR "attention deficit disorder with hyperactivity"[All Fields] OR ("attention"[All Fields] AND "deficit"[All Fields] AND "hyperactivity"[All Fields] AND "disorder"[All Fields]) OR "attention deficit hyperactivity disorder"[All Fields]) OR ("attention deficit disorder with hyperactivity"[MeSH Terms] OR ("attention"[All Fields] AND "deficit"[All Fields] AND "disorder"[All Fields] AND "hyperactivity"[All Fields]) OR "attention deficit disorder with hyperactivity"[All Fields] OR "adhd"[All Fields])) AND (pursuit[All Fields] OR pursuits[All Fields] OR ("pursuit, smooth"[MeSH Terms] OR ("pursuit"[All Fields] AND "smooth"[All Fields]) OR "smooth pursuit"[All Fields] OR ("smooth"[All Fields] AND "pursuit"[All Fields])) OR ("pursuit, smooth"[MeSH Terms] OR ("pursuit"[All Fields] AND "smooth"[All Fields]) OR "smooth pursuit"[All Fields] OR ("smooth"[All Fields] AND "pursuits"[All Fields]) OR "smooth pursuits"[All Fields])) | 2225 |
| (("attention deficit disorder with hyperactivity"[MeSH Terms] OR ("attention"[All Fields] AND "deficit"[All Fields] AND "disorder"[All Fields] AND "hyperactivity"[All Fields]) OR "attention deficit disorder with hyperactivity"[All Fields] OR ("attention"[All Fields] AND "deficit"[All Fields] AND "hyperactivity"[All Fields] AND "disorder"[All Fields]) OR "attention deficit hyperactivity disorder"[All Fields]) OR ("attention deficit disorder with hyperactivity"[MeSH Terms] OR ("attention"[All Fields] AND "deficit"[All Fields] AND "disorder"[All Fields] AND "hyperactivity"[All Fields]) OR "attention deficit disorder with hyperactivity"[All Fields] OR "adhd"[All Fields])) AND (oculomotor[All Fields] OR (oculomotor[All Fields] AND ("abnormalities"[Subheading] OR "abnormalities"[All Fields] OR "congenital abnormalities"[MeSH Terms] OR ("congenital"[All Fields] AND "abnormalities"[All Fields]) OR "congenital abnormalities"[All Fields])) OR (oculomotor[All Fields] AND problem[All Fields]) OR (oculomotor[All Fields] AND deficit[All Fields])) | 1396 |
| (("attention deficit disorder with hyperactivity"[MeSH Terms] OR ("attention"[All Fields] AND "deficit"[All Fields] AND "disorder"[All Fields] AND "hyperactivity"[All Fields]) OR "attention deficit disorder with hyperactivity"[All Fields] OR ("attention"[All Fields] AND "deficit"[All Fields] AND "hyperactivity"[All Fields] AND "disorder"[All Fields]) OR "attention deficit hyperactivity disorder"[All Fields]) OR ("attention deficit disorder with hyperactivity"[MeSH Terms] OR ("attention"[All Fields] AND "deficit"[All Fields] AND "disorder"[All Fields] AND "hyperactivity"[All Fields]) OR "attention deficit disorder with hyperactivity"[All Fields] OR "adhd"[All Fields])) AND (fixation[All Fields] OR (("eye"[MeSH Terms] OR "eye"[All Fields]) AND fixation[All Fields])) | 5159 |
| **Scopus** |  |
| Attention deficit hyperactivity disorder OR ADHD AND saccade OR pursuit OR oculomotor OR eye fixation | 945 |
| attention AND deficit AND hyperactivity AND disorder OR adhd AND saccadic OR antisaccade OR smooth AND pursuit OR pursuits OR oculomotor OR eye AND fixation | 144 |
| saccadic OR antisaccade OR saccade OR smooth AND pursuit OR pursuits OR oculomotor OR eye AND fixation AND attention AND deficit AND hyperactivity AND disorder OR adhd AND ( LIMIT-TO ( DOCTYPE , "ar" ) ) AND ( LIMIT-TO ( ACCESSTYPE(OA) ) ) | 171 |
| saccadic OR antisaccade OR saccade OR smooth AND pursuit OR pursuits OR oculomotor OR eye AND fixation AND attention AND deficit AND hyperactivity AND disorder OR adhd AND ( LIMIT-TO ( DOCTYPE , "ar" ) ) AND ( LIMIT-TO ( ACCESSTYPE(OA) ) ) | 553 |
| **CNHAL Complete** |  |
| TX ( attention deficit hyperactivity disorder or adhd ) AND TX ( saccade OR oculomotor OR pursuit OR eye fixation ) | 268 |
| TX ( attention deficit hyperactivity disorder OR ADHD ) AND TX saccade OR TX oculomotor OR TX pursuit OR TX eye fixation | 2336 |
| TX ( attention deficit hyperactivity disorder or adhd ) AND TX saccadic eye movement OR TX smooth pursuit | 699 |
| ( saccade OR saccadic OR oculomotor OR smooth pursuit OR eye fixation ) AND ( attention deficit hyperactivity disorder or adhd ) | 43 |
| **Web of Science** |  |
| (ALL=( attention deficit hyperactivity disorder OR ADHD ) AND ALL = ( saccade OR saccadic OR saccadic eye movement OR oculomotor OR pursuit OR smooth pursuit OR eye fixation )) AND LANGUAGE: (English) AND DOCUMENT TYPES: (Article) | 194 |
| (ALL= ( saccade OR saccadic OR saccadic eye movement OR smooth pursuits OR fixation) AND ALL= (attention deficit hyperactivity disorder OR ADHD)) AND LANGUAGE: (English) AND DOCUMENT TYPES: (Article) | 176 |
| (ALL= (attention deficit hyperactivity disorder OR ADHD) AND ALL= ( saccade OR saccadic OR saccadic eye movement OR saccades )) AND LANGUAGE: (English) AND DOCUMENT TYPES: (Article) | 113 |
| (ALL= (attention deficit hyperactivity disorder OR ADHD) AND ALL= ( smooth pursuit OR pursuit OR pursuits)) AND LANGUAGE: (English) AND DOCUMENT TYPES: (Article) | 43 |
| (ALL= (attention deficit hyperactivity disorder OR ADHD) AND ALL= ( smooth pursuit OR pursuit OR pursuits)) AND LANGUAGE: (English) AND DOCUMENT TYPES: (Article) | 77 |
| (ALL= (attention deficit hyperactivity disorder OR ADHD ) AND ALL= ( fixation OR eye fixation OR visual fixation)) AND LANGUAGE: (English) AND DOCUMENT TYPES: (Article) | 91 |
| **BMJ** |  |
| attention deficit hyperactivity disorder OR ADHD AND saccade OR oculomotor OR pursuit OR eye fixation | 9 |
| attention deficit hyperactivity disorder OR ADHD AND saccade OR saccadic OR saccadic eye movement OR saccades | 9 |
| attention AND deficit AND hyperactivity AND disorder OR adhd AND pursuit OR smooth pursuit OR pursuits OR smooth pursuits | 31 |
| attention deficit hyperactivity disorder OR ADHD AND oculomotor OR oculomotor abnormalities | 15 |
| attention deficit hyperactivity disorder OR ADHD AND eye fixation OR fixation | 350 |
